# Estimating Prevalence and Characteristics of Statin Intolerance among High and Very High Cardiovascular Risk Patients in Germany (2017 to 2020)

**DOI:** 10.3390/jcm12020705

**Published:** 2023-01-16

**Authors:** Klaus G. Parhofer, Anastassia Anastassopoulou, Henry Calver, Christian Becker, Anirudh S. Rathore, Raj Dave, Cosmin Zamfir

**Affiliations:** 1 Ludwig Maximilians University, Medical Clinic IV, Großhadern, 81377 Munich, Germany; 2 Daiichi Sankyo Europe GmbH, Zielstattstraße 48, 81379 Munich, Germany; 3 IQVIA, London W2 1AF, UK; 4 Daiichi Sankyo Germany GmbH, Zielstattstraße 48, 81379 Munich, Germany; 5 IQVIA, Bangalore 560103, India; 6 IQVIA, 60549 Frankfurt, Germany

**Keywords:** statin intolerance, prevalence, lipid-lowering therapy, cardiovascular risk, advanced analytics, supervised machine learning

## Abstract

Statin intolerance (SI) (partial and absolute) could lead to suboptimal lipid management. The lack of a widely accepted definition of SI results into poor understanding of patient profiles and characteristics. This study aims to estimate SI and better understand patient characteristics, as reflected in clinical practice in Germany using supervised machine learning (ML) techniques. This retrospective cohort study utilized patient records from an outpatient setting in Germany in the IQVIA™ Disease Analyzer. Patients with a high cardiovascular risk, atherosclerotic cardiovascular disease, or hypercholesterolemia, and those on lipid-lowering therapies between 2017 and 2020 were included, and categorized as having “absolute” or “partial” SI. ML techniques were applied to calibrate prevalence estimates, derived from different rules and levels of confidence (high and low). The study included 292,603 patients, 6.4% and 2.8% had with high confidence absolute and partial SI, respectively. After deploying ML, SI prevalence increased approximately by 27% and 57% (*p* < 0.00001) in absolute and partial SI, respectively, eliciting a maximum estimate of 12.5% SI with high confidence. The use of advanced analytics to provide a complementary perspective to current prevalence estimates may inform the identification, optimal treatment, and pragmatic, patient-centered management of SI in Germany.

## 1. Introduction

The 2019 European Society of Cardiology (ESC)/European Atherosclerosis Society (EAS) guidelines on the management of dyslipidemia focus on risk-adapted low-density lipoprotein cholesterol (LDL-C) reduction as a primary strategy [1]. Despite treatment with oral lipid- lowering therapies (LLTs), up to 80% of patients with hypercholesterolemia and mixed dyslipidemia across Europe do not reach guideline-recommended LDL-C goals [1,2,3]. Germany has adapted lipid management goals as outlined in the ESC/EAS guidelines, however, real-world evidence from Germany (2017) indicates that only 36.3% of high and very high CV risk patients seem to be treated with LLTs [4].

Although statin therapy is the accepted primary pharmacological approach for LDL-C reduction, non-adherence to treatment and discontinuation of statin therapy are current problems worldwide [5]. A frequently noted complaint among statin users is statin-associated muscle symptoms (SAMS). Recent updates from the National Lipid Association (NLA) classify SAMS as myalgia, myopathy, myositis, and myonecrosis (including rhabdomyolysis) [6,7]. Besides SAMS, headache, dyspepsia, nausea, alopecia, and erectile dysfunction are a few other reported side effects that can affect a patient’s quality of life [6].

There is no widely accepted definition of statin intolerance (SI), and those available have changed over the years [8]. The NLA in the US defines SI as the inability to tolerate at least two statins, one at the lowest starting daily dose and another at any daily dose, due to either objectionable symptoms or abnormal laboratory determinations that are temporally related to statin treatment and reversible upon statin discontinuation [8,9]. Moreover, the International Lipid Expert Panel (ILEP) definition includes the resolution of symptoms or changes in biomarkers or even significant improvement with dose reduction or withdrawal of treatment [8,10]. The EAS defines SI as an assessment of SAMS that includes the nature of muscle symptoms, increased creatine kinase levels, and their temporal association with statin therapy initiation, and statin therapy suspension and rechallenge [8,11]. Although these are valid definitions, in clinical practice, the manifestations and management of statin intolerance may differ, making it difficult to describe a unanimous condition and a single-point estimate of prevalence. Further, studies have explored additional classifications of SI, namely total vs. partial SI. INTERCATH an observational cohort study had used these classifications of SI to quantify the target populations for bempedoic acid and proprotein convertase subtilisin/kexin type 9 (PCSK9) inhibitors. Treatment goal of the study was to achieve the LDL-C reduction in sub-population of patients with partial and total SI [12].

The prevalence of SI is reported as overestimated worldwide, thus outlining an important clinical challenge for careful assessment and optimal treatment [5]. A recent meta- analysis estimated the overall prevalence of SI at 9.1%. Lower prevalence rates were estimated when the NLA, ILEP, and EAS criteria were applied, as they apply a strict definition (7.0%, 6.7%, and 5.9%, respectively) [5]. Several factors play a role in SI, including advanced age, female gender, race, obesity, diabetes mellitus, hypothyroidism, chronic liver failure, and renal failure [5]. Concurrent use of antiarrhythmic agents and calcium-channel blockers, alcohol use, and high statin dosing are a few other key factors associated with a higher risk of SI [5].

With large administrative datasets available for healthcare research, a possible way to improve SI prevalence estimates is to apply machine learning (ML), which is increasingly being utilized in the modeling of epidemiologic data [13]. This technique allows one to establish a relationship between features and algorithm outputs based on the analyzed dataset [14]. Supervised learning in ML techniques allows the making of predictions from identified data based on features analyzed [14]. Supervised models predict an outcome, analogous to predictive modeling using regression [13].

The current management of SI includes statin-switching, dose-lowering, and intermittent dosing [8]. In addition, combination therapies involving non-statin lipid-lowering drugs, such as ezetimibe, proprotein convertase subtilisin-kexin type 9 inhibitors, bempedoic acid, with or without statins, are useful alternative options for the treatment of SI. In patients who cannot tolerate even the lowest dose of statins, a non-statin regimen is recommended to achieve LDL-C reduction [15].

This study aims to describe the current prevalence of SI based on a large representative real-world cohort of outpatients from Germany. Starting out by applying SI definitions used in clinical practice in Europe to the Electronic Medical Records (EMR) (IQVIA^TM^ Disease Analyzer) data set, estimates were enhanced by applying ML to predict SI in uncertain patients to further improve confidence in prevalence estimates.

With this study, we aim to estimate the current SI prevalence using real-world data and better understand patient characteristics in Germany using advanced analytics. The results may inform about the diagnosis and optimal treatment of SI in Germany.

## 2. Materials and Methods

### 2.1. Study Design

This was a retrospective study based on a cohort of patients on LLT in the IQVIA^TM^ Disease Analyzer from March 2017 to March 2020.

The IQVIA™ Disease Analyzer is based on a representative sample of more than 3300 resident physicians in Germany (as of February 2020) equipped with office-based IT systems [16,17]. The data source contains daily-based anonymized records of treatment-relevant data of statutory health insurance and private health insurance patients. This allows a detailed consideration of the therapeutic and diagnostic behavior of physicians (in retail), as well as a longitudinal view of anonymized patients with their diagnoses and treatments. Unlike in other data sources, outpatient diagnoses are recorded daily (by date of visit).

Patients were included in the study if they were aged at least 18 years or more on the index date with at least one consultation during the selection period (March 2019 to March 2020). Moreover, patients were included if they were on LLTs from March 2017 to March 2020 and had been identified to have hypercholesterolemia or atherosclerotic cardiovascular disease (ASCVD) or high CV risk during the selection period or in the past.

Hypercholesterolemia, LLTs, ASCVD, and high CV risk patients were defined based on Anatomical Therapeutic Chemical (ATC) classes or International Classification of Diseases (ICD)-10 codes (Table S1 for more details).

The index date was defined by the latest prescription of statins for patients actively on treatment medication during the study period. For patients who were on non-statin LLTs during the study period, the index date was defined based on the latest prescription of non-statin LLTs.

Baseline characteristics, comorbidities, concomitant medications, and SI events were assessed during a maximum two-year lookback period, with the earliest possible date being March 2017. The lookback period was based on the index date.

### 2.2. Statin Intolerance

To identify SI, a variety of different events that can signal potential SI were analyzed. The events included statin down-titration (same and different molecule), statin switch/multiple statins (without up- or down-titration), statin discontinuation, intermittent dosing, low-dose statin use, documented SI/toxicity/allergy, and the presence of SAMS—derived from clinical practice in Europe and reflecting the definitions of various societies [18,19] (Table S2 for more details). These events were used later to create rules for the categorization of patients as having SI or not. Final SI definitions were derived based on different combinations of SI events/conditions (Table 1).

We applied two separate classifications of SI, as reported by others [12], in order to differentiate between patients who permanently discontinued statins and patients who exhibited certain SI characteristics but had not completely discontinued statins during the study period:

- Absolute SI: Patients with a history of SI events (Table 1) who permanently discontinued statin use.
- Partial SI: Patients with a history of SI events (Table 1) who were either actively on statins during the selection period (March 2019 to March 2020) or did not have a gap of >180 days from the latest statin prescription and the end of the study period (March 2020).

In addition, the two separate classes of SI were classified into two groups: high and low confidence levels, based on the level of confidence for the specific rules and driven by the stronger association with SI as a clinical syndrome (Table 1) (Tables S5 and S6 for more details).

### 2.3. Supervised Machine Learning Prevalence Estimates

**Figure 1:**
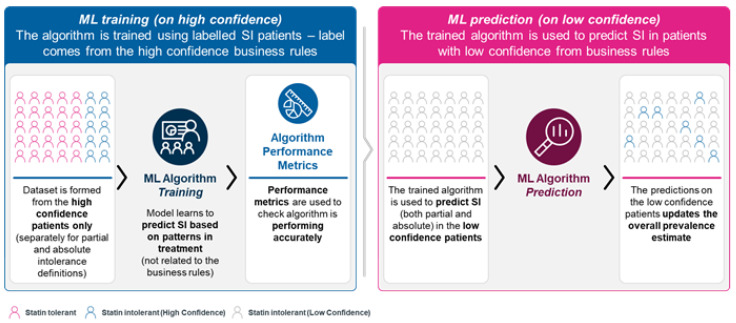
Machine learning algorithm training. ML, machine learning; SI, statin intolerance.

Supervised ML was used to refine the rules of SI prevalence estimate based on the EMR dataset and find the most important features in predicting SI [13,20], as illustrated in Figure 1. An advantage of using ML over traditional regression-based approaches is that previously unknown complex variable interactions and non-linear relationships can also be considered. Two classification models were trained. First on group 1, to differentiate “high confidence absolute intolerant patients versus tolerant patients” and second on group 2, to differentiate “high-confidence partial intolerant patients versus tolerant patients”. The two trained models were used to run a refined classification on low confidence absolute intolerant patients (model 1) and low confidence partial intolerant patients (model 2) respectively, in order to revise the estimate for SI prevalence. SHapley Additive exPlanations (SHAP) feature importance analysis was used to quantify the contributions of the most important features the model uses to predict SI in patients [21].

#### 2.3.1. Training Data Definitions

The models were trained using the same cohorts selected from the EMR IQVIA^TM^ Disease Analyzer dataset. The absolute intolerant dataset consists of all high confidence absolute intolerant patients, along with a random sample of 50,000 tolerant patients. The partial intolerant dataset consists of all high confidence partial intolerant patients, along with a random sample of 50,000 tolerant patients.

#### 2.3.2. Feature Engineering

Features were created from patient-level data based on the presence, frequency, mean values of diagnosis ICD codes, ATC classification codes, and test results. Appropriate ICD codes were also grouped together to create less-granular features. Demographic features included gender and age of the patient. Other features included the number of physician visits and unique prescriptions (molecules and ATC classes and LLT molecules).

#### 2.3.3. Feature Selection

Features were selected by estimating the mutual information between each feature independently and the target variable (statin intolerant vs. tolerant). Mutual information between two random variables is a non-negative value, which measures the dependency between the variables. It is equal to zero when two random variables are independent, and higher values indicate greater dependency. The function relies on nonparametric methods based on entropy estimation from k-nearest neighbors distances as described in Kraskov A et al. [22] and Ross BC [23]. Both methods are based on the concept first proposed in Kozachenko LF et al. [24]. In our model, 400 features were selected for modeling by taking the features with the highest mutual information score. The estimation method uses a nonparametric method based on entropy estimation from a k-nearest neighbors algorithm [22,23].

#### 2.3.4. Model Selection

Different models such as logistic regression, LGBM, and XGBOOST algorithms were trained on the overall dataset [25,26], with the highest performing one being selected based on F1 score (which is the harmonic mean of precision and recall metrics). The dataset was randomly sampled into four equally sized groups. The model was trained on three of the groupings and tested on the remaining one, four times in each possible train-test combination. Performance metrics were evaluated across splits to ensure model performance. Model performance was considered by measuring the receiver operating characteristic (ROC) curve with the area under the curve score, precision-recall curves (PRC), and F1 score.

#### 2.3.5. Prevalence Estimation

The prevalence estimate was updated by predicting for patients labeled as low confidence (absolute and partial intolerant by SI rules) if they were either intolerant or tolerant. The absolute intolerant model was applied to low confidence absolute intolerant patients and the partial intolerant model to low confidence partial intolerant patients. The prevalence was updated by summing up the new total of intolerant patients based on individual patient-level predictions of the model. The decision boundary threshold of both models was adjusted, so that the precision was equal to the recall score of each.

## 3. Results

**Figure 2:**
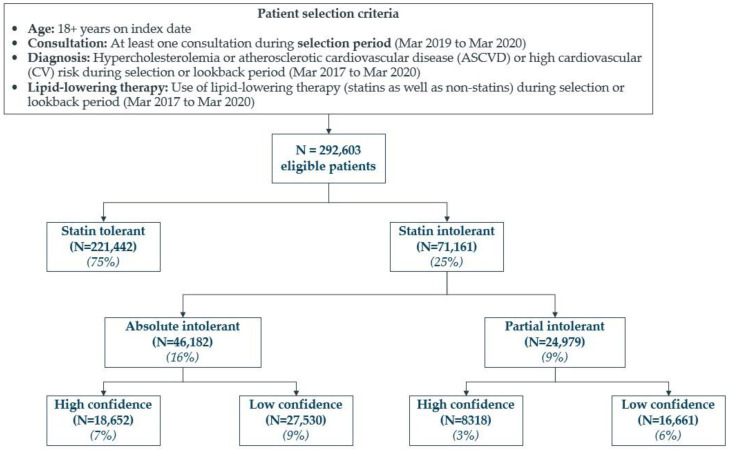
Patient attrition flowchart.

Patient selection criteria and attrition flow for entire study population is summarized in Figure 2. The study included 292,603 patients. Among these, greater number of patients (n = 221,442) were tolerant to statin while n = 71,161 patients were intolerant to statin.

### 3.1. Key Patient and Treatment Characteristics

Key patient and treatment characteristics are summarized in Table 2. Overall, a higher prevalence of SI was observed in patients belonging to the 70+ years age group. The prevalence of absolute SI was higher in females than in males. A ML SHAP analysis was performed to identify key feature importance and key characteristics that help to identify SI patients. Examples are the following: presence of fibrates, use of other lipid-regulators, presence of myalgia, cramps and spasms, total number of prescriptions, and physician visits are significantly higher in ‘absolute’ SI patients than tolerant patients. The ML model used these key predictors to identify low confidence SI patients that could be reclassified as high confidence SI patients. Most preidentified risk factors had a higher prevalence in SI populations compared to tolerant patients. Among the more prevalent risk factors were obesity, hypothyroidism, vitamin D deficiency, and chronic kidney disease. These risk factors also had a higher presence across different intolerant groups.

Simvastatin (47.2%; total) and atorvastatin (36.8%; total) were the most common statins used across both absolute and partial SI patients across all confidence levels. Among statin tolerant patients, simvastatin and atorvastatin were used by 49.6% and 36.9% patients, respectively. Absolute intolerant patients with a high confidence level showed higher non-statin monotherapy (fenofibrate and bezafibrate) use compared to partial SI patients with a high confidence level. Similarly, partially intolerant patients with a high confidence level were using higher (54.0%) ezetimibe as monotherapy or combination with statinscompared to absolute intolerant patients with a high confidence level (21.6%, only). SAMS were consistently observed to occur more frequently in SI groups; the underlying SAMS conditions observed most frequently across confidence levels were myalgia and cramps/spasms. Absolute SI patients exhibited a higher prevalence of known manifestations such as myalgia and cramps/spasms. Drugs that could interact with statins had a slightly higher use among intolerant patients compared to tolerant patients (Table 2).

Detailed patient and treatment characteristics are presented in Table S7.

**Figure 3:**
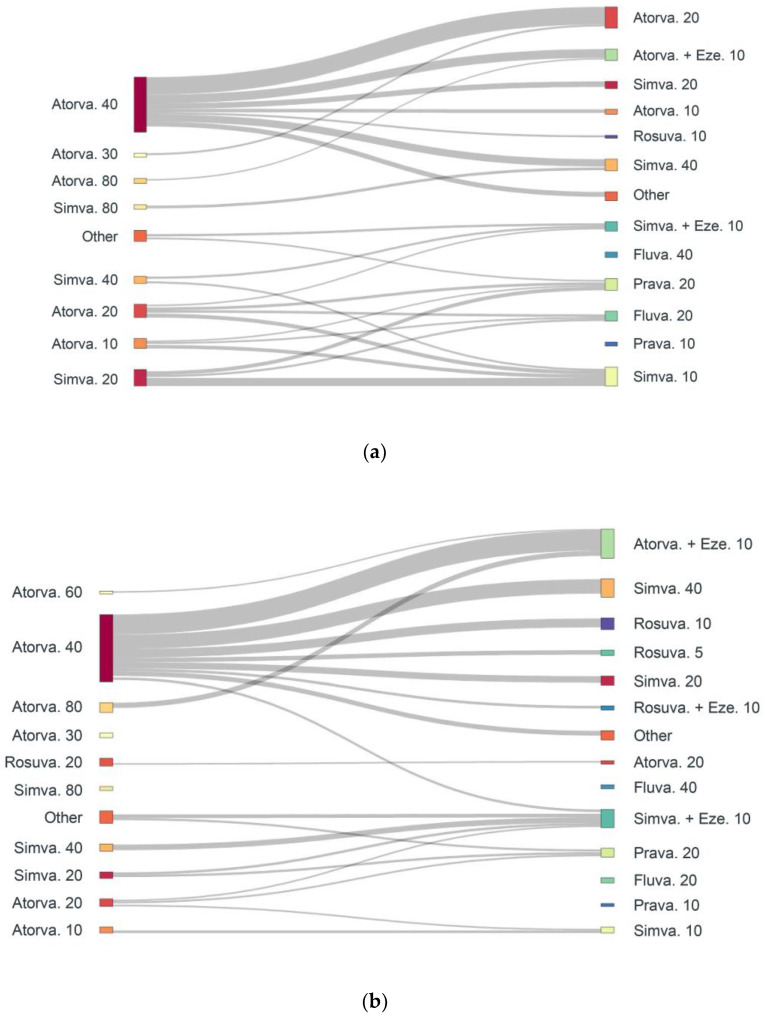
Down-titration in both absolute & partial high confidence SI patients (**a**) Absolute intolerance (1468 down-titrations) 1st statin down-titration event; (**b**) Partial intolerance (6540 down-titrations) 1st statin down-titration event. Atorva, atorvastatin; Eze, ezetimibe; Fluva, fluvastatin; Prava, pravastatin; Rosuva, rosuvastatin; Simva, simvastatin.

**Figure 4:**
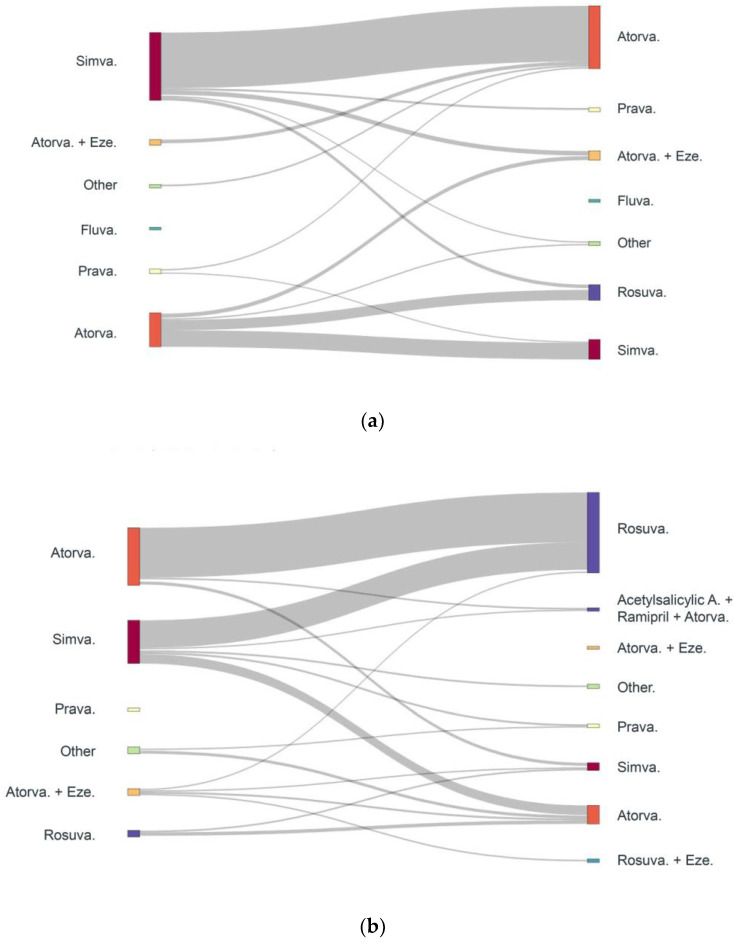
Treatment switches in both absolute & partial high confidence SI patients (**a**) Absolute intolerance (1869 switches) 1st statin switch event; (**b**) Partial intolerance (1848 switches) 1st statin switch event. Atorva, atorvastatin; Eze, ezetimibe; Fluva, fluvastatin; Prava, pravastatin; Rosuva, rosuvastatin; Simva, simvastatin.

Statin down-titration and switches for absolute intolerant and partial intolerant patients are presented in Figure 3 and Figure 4, respectively. Atorvastatin 40 mg was the most frequently down-titrated statin, with patients typically shifting from a high- to medium-intensity dose. Simvastatin to atorvastatin was the most predominant class switch in SI patients, followed by atorvastatin to rosuvastatin. Simvastatin 20 mg to atorvastatin 20 mg represents the highest proportion of switches among intolerant patients, followed by simvastatin 40 mg to atorvastatin 20 mg.

### 3.2. Prevalence Results

Prevalence estimates for SI based on EMR data are presented in Table 3. Among the 292,603 (100%) total eligible patients, the prevalence of tolerant patients was 76.6% (n = 224,112), while the SI prevalence was estimated at a maximum of 24.3% (n = 71,161). The fraction of SI condition among all the ASCVD patients in the study was 24.3%, for the high CV risk patients it was 28.2% and for the hypercholesterolemia patients it was 20.9% (Table S8). The absolute SI prevalence (high confidence) was 6.4%, based on SI rules. However, this was enhanced to 8.1% when supervised ML was applied (~27% increase in patients classified with high confidence). Similarly, partial SI prevalence (high confidence) was 2.8%, based on SI rules, but it was enhanced to 4.4% using supervised ML (~57% increase in patients classified with high confidence). Through supervised ML, the prevalence of high confidence increased by nearly 1.6% in both absolute and partial SI patients, resulting in an overall estimate for high tolerance SI of 12.5%. The overall prevalence estimate remained the same, as the model was trained on high confidence SI patients and applied to low confidence SI patients. The resultant increase of 1.6%, potentially provides a more precise estimate of SI prevalence and helps us to identify a pool of low confidence SI patients that, though do not display specific signs of SI such as down-titration, have characteristics similar to that of high confidence SI patients.

As detailed in the methods (Section Model selection)—model performance was considered by measuring the ROC curve with the area under the curve score, PRC, and F1 score. XGB classifier model was the best performing model. For model 1 and model 2, the area under the ROC curve was 0.91 and 0.90, the area under the PRC curve was 0.61 and 0.53, and the F-score was 0.69 and 0.61, respectively.

## 4. Discussion

The results of this study highlight the challenge of estimating and describing SI among patients with high and very high CV risk in routine clinical practice in Germany. The data were obtained in Germany but probably also hold true in other countries. The identification of pragmatic groups of patients with SI depends upon a range of clinical observations events and characteristics [27]. Statin down-titration, statin switch, statin discontinuation, intermittent dosing, low-dose statin use, documented SI/toxicity/allergy, and the presence of SAMS were events used to define SI—also reflecting definitions of various societies [18,19]. Agreement exists that these events may lead to non-adherence, treatment discontinuation, and often, suboptimal lipid treatment.

The present analysis supports the distinction between partial (i.e., only some types of statins at some doses) and absolute (e.g., all statins at any dose) SI, as also published elsewhere. In a recent meta-analysis, the authors referred to “complete” SI, but this has different definitions in the ILEP and NLA guidelines and is not defined in the EAS guidelines they used [5]. Overall, though they reported a 9.1% prevalence for SI worldwide; randomized controlled trials reported a prevalence below 5% compared to 17% with cohort studies [5]. In our study, the absolute SI prevalence (high confidence) was 6.4%, based on SI rules. However, this prevalence was enhanced to 8.1% when supervised ML was leveraged. These prevalence estimates are very similar to recently published figures on SI prevalence [5]. The contemporary observational cohort study INTERCATH in Germany used simulations to identify the appropriate target population for certain lipid treatments and the related costs generated [12]. In this study, the authors used the concept of partial SI as the inability to tolerate a high-intensity statin and full SI as the inability to tolerate any statin at any dose, which are aligned to ours and previous publications [28].

Additionally, based on the level of confidence SI were classified into high and low confidence levels in our study. This is an innovative way to classify SI, and is a new artificial way invented to assist the SI classification and patient characterization. Validation of the results was performed with qualitative insights from primary market research conducted with consulting external experts (validation data is available upon request) [29].

Regardless of the confidence levels in SI classification, the nocebo effect could be a challenge. Due to the negative expectations of patients about adverse events related to statin use, such patients were more likely to experience the nocebo effect [30]. A systematic review of open-label, blinded phase trials estimated that about 38% to 78% of SAMS-related SI could be attributed to expectation alone/the nocebo effect [27]. As per the self-assessment method for statin side effects or nocebo (SAMSON) trial, 90% of statin complaints were related to the nocebo effect [31]. This finding highlights that patients on statin therapy experienced side effects; however, side effects can be related to the act of taking medications and not essentially to statin use. Physicians should consider prevention and management approaches for the nocebo/drucebo effect at the point of therapy initiation [32]. These approaches are crucial to achieving optimal LLT treatment goals and the management of the CV risk in a large number of patients [32].

There are various risk factors/conditions that may affect the risk of SI. In our study, intolerance was observed more often among women and the elderly. Obesity, hypothyroidism, vitamin D deficiency, and chronic kidney disease were the more prevalent risk factors for the manifestation of SI, aligned with published literature [5]. The current study also revealed antibiotics to be a factor associated with an increased risk of SI, due to a potential drug–drug interaction, as also reported by Fitchett DH et al. [28].

From the physician’s viewpoint, there are several barriers associated with the prescription of and adherence to statin therapies, including partial knowledge, inconsistent clinical guidelines, and a lack of a system to identify the right patients for statin therapy. However, from the patient’s viewpoint, fear of side effects and resistance to taking additional medications are key barriers [33,34]. Hence, there is an opportunity to educate physicians and patients during the treatment journey. A holistic, patient-centric approach is required for the optimal CV risk reduction, continued therapy or alternative drugs and management strategies [32]. The Personalized Lipid Intervention Plan could be utilized for this purpose [35], which together with ML techniques can not only enhance the patients’ education but also the prediction of their behaviour.

### Study Limitations

With the EMR database, the course of treatment can only be tracked longitudinally within the same physician’s office (practice). However, any previous or following diagnosis or treatment by another physician is not recorded. The EMR database only covers outpatient data, while no inpatient data are covered. In addition, outpatient or medical care center (ambulances or MVZ) data are not covered. The results of the analysis were based on the opinions of the panel of general practitioners (GPs) or cardiologists at practice level. In the projection, data for patients presenting to cardiologists (current coverage ~6%) and GPs (current coverage ~3%) were extrapolated separately to the respective specialist populations in Germany. Referrals to specialists or hospitals were documented by physicians, but treatment in other medical practices or hospitals was not recorded. Another limitation is that data on exercise, ethnicity, and family history of SAMS were not included in the study, and these can influence the outcomes of statin therapy and the prevalence estimates of SI indirectly. SI classification approach of high and low confidence levels is an innovative approach but not scientifically quantitative. Although the approach was validated qualitatively with primary market research, even so validation of the approach is considered a limitation for this study. The study estimates SI prevalence based on rules developed using real-world diagnosis and prescription characteristics, and thus cannot differentiate between real and self-opinionated SI prevalence.

## 5. Conclusions

In this study we explored the population of SI patients in Germany using advanced analytics. The derived prevalence estimates and patient characteristics add a complementary point of view to existing knowledge that may inform the diagnosis, optimal treatment, and pragmatic, patient-centered definition of SI.

## Figures and Tables

**Table 1:** Rules for defining statin intolerance by confidence level.

Confidence Level	Absolute SI: If Patients Had History of SI Events and Permanently Discontinued Statin Use	Partial SI: If Patients Had History of SI Events and Are Actively on Statins during the Selection Period and Have Not Discontinued for >180 Days
High	ASCVD and high CV risk patients ONLY ON non-statins	Patients WITHOUT discontinuation of latest statins AND WITH signs of down-titration, multi-statin use, low-dose statin use, SAMS, intermittent dosing, prior discontinuation, or documented SI in notes
	Long-term discontinuation (>180 days) of statin AND signs of down-titration, low-dose statin use, multi-statin use, SAMS, intermittent dosing, documented SI in notes, or prior discontinuation	
Low	Long-term discontinuation AND WITHOUT ANY signs of down-titration, switch, low-dose statin use, SAMS, intermittent dosing, or SI in notes	ASCVD/high CV risk patients on low-intensity statins AND WITHOUT ANY sign of prior discontinuation, down-titration, switch, SAMS, intermittent dosing, or documented SI in notes
		Patients WITHOUT ANY (intermittent dosing OR discontinuation of latest statins), AND WITH signs of down-titration or switch

SI, statin intolerance; SAMS, Statin-associated muscle symptoms.

**Table 2:** Patient and treatment characteristics.

Features	Statin Tolerant	Statin Tolerant	Absolute Statin Intolerant *		Partial Statin Intolerant		Total
			High Confidence	Low Confidence	High Confidence	Low Confidence	
	n = 221,442	n = 71,161	n = 18,652	n = 27,530	n = 8318	n = 16,661	n = 292,603
Age n (%)							
18–30	351 (0.2%)	200 (0.3%)	42 (0.2%)	124 (0.5%)	11 (0.1%)	23 (0.1%)	551 (0.2%)
30–50	7501 (3.4%)	3353 (4.7%)	730 (3.9%)	1729 (6.3%)	344 (4.1%)	550 (3.3%)	10,854 (3.7%)
50–70	84,855 (38.3%)	28,573 (40.2%)	7137 (38.3%)	10,907 (39.6%)	3695 (44.4%)	6834 (41.0%)	113,428 (38.8%)
70+	128,735 (58.1%)	39,035 (54.9%)	10,743 (57.6%)	14,770 (53.7%)	4268 (51.3%)	9254 (55.5%)	167,770 (57.3%)
Gender n (%)							
Female	95,743 (43.2%)	33,591 (47.2%)	8963 (48.1%)	13,876 (50.4%)	3574 (43.0%)	7178 (43.1%)	129,334 (44.2%)
Male	125,302 (56.6%)	37,399 (52.6%)	9653 (51.8%)	13,587 (49.4%)	4724 (56.8%)	9435 (56.6%)	162,701 (55.6%)
Unspecified	397 (0.2%)	171 (0.2%)	36 (0.2%)	67 (0.2%)	20 (0.2%)	48 (0.3%)	568 (0.2%)
Patient subgroups n (%)							
ASCVD	124,937 (56.4%)	40,023 (56.2%)	10,089 (54.1%)	13,405 (48.7%)	5688 (68.4%)	10,841 (65.1%)	164,960 (56.4%)
High CV risk	43,918 (19.8%)	17,224 (24.2%)	5367 (28.8%)	7076 (25.7%)	1541 (18.5%)	3240 (19.4%)	61,142 (20.9%)
Hypercholesterolemia	52,587 (23.7%)	13,914 (19.6%)	3196 (17.1%)	7049 (25.6%)	1089 (13.1%)	2580 (15.5%)	66,501 (22.7%)
Risk factors n (%)							
Obesity	20,877 (9.4%)	6820 (9.6%)	1701 (9.1%)	2583 (9.4%)	813 (9.8%)	1723 (10.3%)	27,697 (9.5%)
Frailty and senility	8802 (4.0%)	2673 (3.8%)	751 (4.0%)	1019 (3.7%)	236 (2.8%)	667 (4.0%)	11,475 (3.9%)
Vitamin D deficiency	38,092 (17.2%)	14,575 (20.5%)	3935 (21.1%)	5233 (19.0%)	1766 (21.2%)	3641 (21.9%)	52,667 (18.0%)
Alcohol-abuse-related conditions	3330 (1.5%)	1094 (1.5%)	275 (1.5%)	491 (1.8%)	88 (1.1%)	240 (1.4%)	4424 (1.5%)
Hypothyroidism	19,512 (8.8%)	6571 (9.2%)	1697 (9.1%)	2486 (9.0%)	792 (9.5%)	1596 (9.6%)	26,083 (8.9%)
Liver disease ^	2336 (1.1%)	871 (1.2%)	264 (1.4%)	326 (1.2%)	88 (1.1%)	193 (1.2%)	3207 (1.1%)
CKD ^^	10,102 (4.6%)	3593 (5.0%)	993 (5.3%)	1394 (5.1%)	376 (4.5%)	830 (5.0%)	13,695 (4.7%)
Treatment usage							
Statins n (%)							
Simvastatin	109,835 (49.6%)	28,390 (39.9%)	7535 (40.4%)	12,719 (46.2%)	2287 (27.5%)	5848 (35.1%)	138,225 (47.2%)
Atorvastatin	81,712 (36.9%)	26,011 (36.6%)	5521 (29.6%)	12,141 (44.1%)	4068 (48.9%)	4282 (25.7%)	107,723 (36.8%)
Rosuvastatin	5757 (2.6%)	1284 (1.8%)	37 (0.2%)	606 (2.2%)	208 (2.5%)	433 (2.6%)	7041 (2.4%)
Non-statins n (%)							
Ezetimibe (Mono or in combination with statins)	21,291 (9.6%)	14,743 (20.7%)	4031 (21.6%)	1402 (5.1%)	4490 (54.0%)	4820 (28.9%)	36,034 (12.3%)
Fenofibrate monotherapy	1329 (0.6%)	933 (1.4%)	933 (5.0%)	0 (0%)	0 (0%)	1200 (7.2%)	2262 (0.8%)
Bezafibrate monotherapy	1107 (0.5%)	801 (1.2%)	746 (4.0%)	55 (0.2%)	0 (0%)	0 (0%)	1908 (0.7%)
SAMS n (%)							
Myalgia	4240 (1.9%)	1882 (2.6%)	800 (4.3%)	356 (1.3%)	330 (4.0%)	396 (2.4%)	6122 (2.1%)
Myositis	76 (0%)	60 (0.1%)	31 (0.2%)	8 (0%)	9 (0.1%)	12 (0.1%)	136 (0%)
Myopathy, unspecified	165 (0.1%)	130 (0.2%)	62 (0.3%)	18 (0.1%)	22 (0.3%)	28 (0.2%)	295 (0.1%)
Cramps/spasms of the muscles	4808 (2.2%)	1864 (2.6%)	769 (4.1%)	330 (1.2%)	326 (3.9%)	439 (2.6%)	6672 (2.3%)
Other adverse events n (%)							
Constipation	12,121 (5.5%)	4408 (6.2%)	1224 (6.6%)	1869 (6.8%)	427 (5.1%)	888 (5.3%)	16,529 (5.6%)
Abdominal pain	11,906 (5.4%)	4750 (6.7%)	1288 (6.9%)	1773 (6.4%)	567 (6.8%)	1122 (6.7%)	16,656 (5.7%)
Flatulence	4181 (1.9%)	1727 (2.4%)	511 (2.7%)	613 (2.2%)	197 (2.4%)	406 (2.4%)	5908 (2.0%)
Nausea and vomiting	8261 (3.7%)	3450 (4.8%)	944 (5.1%)	1422 (5.2%)	376 (4.5%)	708 (4.2%)	11,711 (4.0%)
Gastritis & duodenitis	30,214 (13.6%)	10,731 (15.1%)	2863 (15.3%)	3976 (14.4%)	1328 (16.0%)	2564 (15.4%)	40,945 (14.0%)
Anaphylaxis	5840 (2.6%)	2344 (3.3%)	608 (3.3%)	896 (3.3%)	299 (3.6%)	541 (3.2%)	8184 (2.8%)
Rash and flushing	9108 (4.1%)	3397 (4.8%)	922 (4.9%)	1293 (4.7%)	399 (4.8%)	783 (4.7%)	12,505 (4.3%)
Cognitive impairment	4001 (1.8%)	1323 (1.9%)	357 (1.9%)	509 (1.8%)	129 (1.6%)	328 (2.0%)	5324 (1.8%)
Drug–Drug n (%)							
Antibiotics **	9833 (4.4%)	3615 (5.1%)	1027 (5.5%)	1425 (5.2%)	392 (4.7%)	771 (4.6%)	13,448 (4.6%)
Verapamil, diltiazem	3649 (1.6%)	1353 (1.9%)	381 (2.0%)	484 (1.8%)	156 (1.9%)	332 (2.0%)	5002 (1.7%)
Amiodarone	4557 (2.1%)	1596 (2.2%)	382 (2.0%)	550 (2.0%)	215 (2.6%)	449 (2.7%)	6153 (2.1%)

ASCVD, atherosclerotic cardiovascular disease; CKD, chronic kidney disease; SAMS: statin-associated muscle symptoms. *: For absolute SI patients latest LLTs usage before permanent discontinuation was captured in the table (during March 2017 to March 2019 i.e., the period of their past statins medication); **: Antibiotics include erythromycin, telithromycin, clarithromycin; ^ CKD defined by ICD codes N18.3, N18.4, N18.5 representing moderate to severe chronic kidney disease; ^^ liver disease defined by ICD codes K70 to K77—includes conditions such as toxic liver disease, liver failure, chronic hepatitis, and cirrhosis of liver.

**Table 3:** Statin intolerance prevalence estimates based on EMR data (by confidence level).

Prevalence Estimates	Total Universe	Statin Tolerant	Statin Intolerant			
			By Confidence Level			
		Total	Absolute Statin Intolerant		Partial Statin Intolerant	
			High Confidence	Low Confidence	High Confidence	Low Confidence
SI rules (EMR)	292,603 (100%)	224,112 (76.6%)	18,652 (6.4%)	27,530 (9.4%)	8318 (2.8%)	16,661 (5.7%)
SI rules + supervised ML (EMR)			23,718 (8.1%)	22,464 (7.7%)	12,836 (4.4%)	12,143 (4.1%)

EMR, electronic medical record; ML, machine learning; SI, statin intolerance.

## Data Availability

The data that support the study findings presented in this manuscript are available from the corresponding author, upon reasonable request. The data are not publicly available due to confidentiality purposes.

## Supplementary Materials

The following supporting information can be downloaded at: https://www.mdpi.com/article/10.3390/jcm12020705/s1, Table S1: Definition of LLTs, hypercholesterolemia, ASCVD, and high CV risk; Table S2: Definition of statin intolerance (SI) events; Table S3: Definition of lipid-lowering therapies (LLTs); Table S4: Classification of statin intensity [36]; Table S5: Absolute statin intolerance—SI rules and cohort segments by level of confidence; Table S6: Partial statin intolerance—SI rules and cohort segments by level of confidence; Table S7: Patient and treatment characteristics (age, gender, patient subgroups, and risk); Table S8: Statin intolerance prevalence estimates based on EMR data (by risk factors).
