# Supplementary material for: Estimating Prevalence and Characteristics of Statin Intolerance among High and Very High Cardiovascular Risk Patients in Germany (2017 to 2020)
Source: J Clin Med. 2023 Jan 16;12(2):705. doi: 10.3390/jcm12020705 (PMC9864390; doi:10.3390/jcm12020705)
Supplement: Supplementary file 1 [file jcm-12-00705-s001.zip › jcm-2100318-supplementary.pdf]

Supplementary Materials

Table S1. Definition of LLTs, hypercholesterolemia, ASCVD, and high CV risk.

Segment	Definitions
Lipid-lowering therapies (LLTs)	Defined based on Anatomical Therapeutic Chemical (ATC) classes:<br>• C10A (cholesterol- and triglyceride-regulating preparations)<br>• C10C (lipid-regulators in combination with other lipid-regulators)<br>• C11A (lipid-regulating cardiovascular multitherapy combination products)
Hypercholesterolemia	Defined by a confirmed diagnosis based on ICD-10 codes:<br>• E78.0 (pure hypercholesterolemia)<br>• E78.2 (mixed hyperlipidemia)<br>• E78.4 (other hyperlipidemias)<br>• E78.5 (hyperlipidemia, unspecified)<br>• E78.9 (disorder of lipoprotein metabolism unspecified)<br>• E78.8 (other disorders of lipoprotein metabolism)
Atherosclerotic cardiovascular disease (ASCVD)	Defined by a confirmed diagnosis based on ICD-10 codes:<br>• I20–I25 (coronary heart disease and coronary artery disease)<br>• I63, I64, G45 cerebrovascular disease<br>• I70–I74 atherosclerosis, aortic aneurysm, and peripheral vascular disease<br>• Z955 presence of coronary angioplasty implant and graft<br>• Z951 presence of aortocoronary bypass graft (presence of aortocoronary bypass graft)
High CV risk	Was defined by a confirmed diagnosis of any of the ICD-10 codes and conditions below:<br>• E10 (type 1 diabetes) or E11 (type 2 diabetes), female aged >65 years, male aged >60 years<br>• LDL-cholesterol value >190 mg/dL or total cholesterol value >310 mg/dL<br>• N18.3, N18.4, N18.5 moderate-to-severe chronic kidney disease<br>*Note: blood pressure values and smoking status were not used to define high CV risk.*

ICD, International Classification of Diseases; ASCVD, atherosclerotic cardiovascular disease; CV, cardiovascular; LLTs, lipid-lowering therapies.

Table S2. Definition of statin intolerance (SI) events.

Event/s	Definition
Statin down-titration (same molecule)	A reduction in strength of consecutive statin prescriptions if the same molecule.
Statin down-titration (different molecule)	A reduction in dosage intensity (based on Table S4), for consecutive statin prescriptions.
Statin switch/multiple statins (without up- or down- titration)	A switch of molecule for consecutive statin prescriptions, if the same dosage intensity (based on Table S4).
Statin discontinuation	No statin prescriptions for more than 180 days over the median treatment length for given pack.
Intermittent dosing	No statin prescriptions for over twice the median treatment length of pack.
Low-dose statin use	Low-dose statin therapy was defined as an average daily dose of rosuvastatin 5 mg or lower, atorvastatin 10 mg or lower, simvastatin 10 mg or lower, lovastatin 20 mg or lower, pravastatin 40 mg or lower, fluvastatin 40 mg or lower, or pitavastatin 2 mg or lower.
Documented SI/toxicity/allergy	ICD codes:<br>• T466 (poisoning by antihyperlipidemic and anti-arteriosclerosis drugs)<br>• Y526 (adverse effects in therapeutic use: antihyperlipidemic and ant arteriosclerotic drugs)<br>• T887 (unspecified undesirable side effect of a medicine or drug)
Presence of SAMS	SAMS-related ICD codes:<br>• M628 (rhabdomyolysis)<br>• M608 (other myositis)<br>• M791 (myalgia)<br>• M609 (myositis)<br>• G720 (drug-induced myopathy)<br>• G728 (other specified myopathies)<br>• G722 (myopathy caused by other toxic agents)<br>• G729 (myopathy, unspecified)<br>• R25.2 (cramps and spasms of the muscles)

ICD, International Classification of Diseases; SI, statin intolerance; SAMS, statin-associated muscle symptoms.

Table S3. Definition of lipid-lowering therapies (LLTs).

WHO-ATC	Substance	Group
C10AA01	Simvastatin	Statins
C10AA02	Lovastatin	
C10AA03	Pravastatin	
C10AA04	Fluvastatin	
C10AA05	Atorvastatin	
C10AA07	Rosuvastatin	
C10AA08	Pitavastatin	
C10AX09	Ezetimibe	Ezetimibe
C10BA02	Simvastatin + ezetimibe	Statins + Ezetimibe FDC
C10BA05	Atorvastatin + ezetimibe	
C10BA06	Rosuvastatin + ezetimibe	
C10AX13	Evolocumab	PCSK9 inhibitors
C10AX14	Alirocumab	
C10AX16	Inclisiran	

FDC, fixed dose combination; PCSK9, proprotein convertase subtilisin/kexin type 9; WHO-ATC, World Health Organization Anatomical Therapeutic Chemical code.

Table S4. Definition of lipid-lowering therapies (LLTs).

Substance	Low-intensity (LDL-C reduction <30%)	Moderate-intensity (LDL-C reduction 30% to <50%)	High-intensity (LDL-C reduction >50%)
Atorvastatin	-	<30 mg	≥30 mg
Fluvastatin	<60 mg	≥60 mg	-
Lovastatin	<30 mg	≥30 mg	-
Pitavastatin	<1.5 mg	≥1.5 mg	-
Pravastatin	<30 mg	≥30 mg	-
Rosuvastatin	-	<15 mg	≥15 mg
Simvastatin	<15 mg	15–60 mg	≥60 mg

LDL-C, low-density lipoprotein cholesterol.
The classification of statin intensity is based on Fox et al. 2017 (Table S1) [36], which was used to identify up- and down-titration events only.
Eligible patients were classified into three key cohorts for analysis of SI (Table S5):

- Cohort A: Patients not taking lipid-lowering medications during the selection period (March 2019 to March 2020), but who had previously taken statins (March 2017 to March 2019).
- Cohort B: Patients with ASCVD and high CV risk who were only on non-statin lipid-lowering medications during the selection period.
- Cohort C: Patients on ≥1 statins during the selection period (March 2019 to March 2020).

Table S5. Absolute statin intolerance – SI rules and cohort segments by level of confidence.

Confidence	Cohort	Segment	Rule
**High**	B	All patients	Patients **ONLY ON** non-statins
	A	All patients	Long-term discontinuation **AND** down-titration (different molecule) or switch from atorvastatin/simvastatin to rosuvastatin 5 mg/pravastatin/fluvastatin
	A	• ASCVD/High CV risk<br>• Low-intensity statin as latest prescription	Long-term discontinuation **AND** low-dose statin as the latest prescription
	A	• ASCVD/High CV risk<br>• **No** low-intensity statin latest prescription	Long-term discontinuation **AND** history of any SI event, including documented SI in notes or statin down-titration (same or different molecule) or statin switch or SAMS or intermittent dosing
	A	**No** ASCVD/High CV risk patients	Long-term discontinuation **AND** history of any SI event, including documented SI in notes or statin down-titration (same or different molecule) or statin switch or SAMS or intermittent dosing
	C	• ASCVD/High CV risk<br>• Low-intensity statin as latest prescription	Patients with discontinuation for latest statins **AND** history of documented SI in notes **OR** statin down-titration (same or different molecule) **OR** statin switch
**Moderate**	A	• ASCVD/High CV risk<br>• **No** low-intensity statin as latest prescription	Long-term discontinuation **AND** ***SI events limited to only*** (SAMS **OR** intermittent dosing **OR** prior discontinuation)
	A	**No** ASCVD/High CV risk	Long-term discontinuation **AND** ***SI events limited to only*** (SAMS **OR** intermittent dosing **OR** prior discontinuation)
	C	• ASCVD/High CV risk<br>• **No** low-intensity statin as latest prescription	Patients with discontinuation for latest statins **AND** history of documented SI in notes **OR** statin down-titration (same or different molecule) **OR** statin switch
	C	**No** ASCVD/High CV risk	Patients with discontinuation for latest statins **AND** history of documented SI in notes **OR** statin down-titration (same or different molecule) **OR** statin switch
**Low**	A	**No** ASCVD/High CV risk patients	Long-term discontinuation **AND WITHOUT ANY** history of any SI event, including documented SI in notes **OR** statin down-titration (same or different molecule) **OR** statin switch or SAMS **OR** intermittent dosing **OR** prior discontinuation
	A	• ASCVD/High CV risk<br>• **No** low-intensity statin as latest prescription	Long-term discontinuation **AND WITHOUT ANY** history of any SI event, including documented SI in notes **OR** statin down-titration (same or different molecule) **OR** statin switch or SAMS **OR** intermittent dosing **OR** prior discontinuation

ASCVD, atherosclerotic cardiovascular disease; CV, cardiovascular; SAMS, statin-associated muscle symptoms; SI, statin intolerance.

Table S6. Partial statin intolerance – SI rules and cohort segments by level of confidence.

Confidence	Cohort	Segment	Rule
**High**	C	All patients	Patients **WITHOUT** discontinuation for latest statins **AND** with down-titration (different molecule) or switch from atorvastatin/simvastatin to rosuvastatin 5 mg/pravastatin/fluvastatin
	C	• ASCVD/High CV risk<br>• Low-intensity statin as latest prescription	Patients **WITHOUT** discontinuation for latest statins **AND** **WITH** history of documented SI in notes **OR** statin down-titration (same or different molecule) **OR** statin switch
**Moderate**	C	• ASCVD/High CV risk<br>• Low-intensity statin as latest prescription	Patients **WITHOUT** discontinuation for latest statins **AND** ***SI events limited to only*** (SAMS **OR** intermittent dosing **OR** prior discontinuation)
	C	• ASCVD/High CV risk<br>• **No** low-intensity statin latest prescription	Patients with only intermittent dosing of latest statins **AND** history of documented SI in notes **OR** statin down-titration (same or different molecule) **OR** statin switch
	C	**No** ASCVD/High CV risk	Patients with only intermittent dosing of latest statins **AND** history of documented SI in notes **OR** statin down-titration (same or different molecule) **OR** statin switch
**Low**	C	• ASCVD/High CV risk<br>• Low-intensity statin as latest prescription	Low-intensity stain usage **AND WITHOUT ANY history of a SI event, including** documented SI in notes **OR** statin down-titration (same or different molecule) **OR** statin switch **OR** SAMS **OR** intermittent dosing **OR** prior discontinuation
	C	• ASCVD/High CV risk<br>• **No** low-intensity statin latest prescription	Patients **WITHOUT ANY** intermittent dosing **OR** discontinuation of latest statins **WITH** history of documented SI in notes **OR** statin down-titration (same or different molecule) **OR** statin switch
	**C**	**No** ASCVD/High CV risk	Patients **WITHOUT ANY** intermittent dosing OR discontinuation of latest statins **AND WITH** history of documented SI in notes **OR** statin down-titration (same or different molecule) **OR** statin switch

ASCVD, atherosclerotic cardiovascular disease; CV, cardiovascular: SAMS, statin- associated muscle symptoms; SI, statin intolerance.

Table S7. Patient and treatment characteristics (age, gender, patient subgroups, and risk).

Features	Statin tolerant	Statin intolerant	Absolute statin intolerant		Partial statin intolerant		Total
			High confidence	Low confidence	High confidence	Low confidence	
	n = 221,442	n = 71,161	n = 18,652	n = 27,530	n = 8,318	n = 16,661	n = 292,603
**Age**							
18–30	351 (0.2%)	200 (0.3%)	42 (0.2%)	124 (0.5%)	11 (0.1%)	23 (0.1%)	551 (0.2%)
30–50	7,501 (3.4%)	3,353 (4.7%)	730 (3.9%)	1,729 (6.3%)	344 (4.1%)	550 (3.3%)	10,854 (3.7%)
50–70	84,855 (38.3%)	28,573 (40.2%)	7,137 (38.3%)	10,907 (39.6%)	3,695 (44.4%)	6,834 (41.0%)	113,428 (38.8%)
70+	128,735 (58.1%)	39,035 (54.9%)	10,743 (57.6%)	14,770 (53.7%)	4,268 (51.3%)	9,254 (55.5%)	167,770 (57.3%)
**Gender**							
Female	95,743 (43.2%)	33,591 (47.2%)	8,963 (48.1%)	13,876 (50.4%)	3,574 (43.0%)	7,178 (43.1%)	129,334 (44.2%)
Male	125,302 (56.6%)	37,399 (52.6%)	9,653 (51.8%)	13,587 (49.4%)	4,724 (56.8%)	9,435 (56.6%)	162,701 (55.6%)
Unspecified	397 (0.2%)	171 (0.2%)	36 (0.2%)	67 (0.2%)	20 (0.2%)	48 (0.3%)	568 (0.2%)
ASCVD	124,937 (56.4%)	40,023 (56.2%)	10,089 (54.1%)	13,405 (48.7%)	5,688 (68.4%)	10,841 (65.1%)	164,960 (56.4%)
High CV risk	43,918 (19.8%)	17,224 (24.2%)	5,367 (28.8%)	7,076 (25.7%)	1,541 (18.5%)	3,240 (19.4%)	61,142 (20.9%)
Hypercholesterolemia	52,587 (23.7%)	13,914 (19.6%)	3,196 (17.1%)	7,049 (25.6%)	1,089 (13.1%)	2,580 (15.5%)	66,501 (22.7%)
**Risk factors**							
Obesity	20,877 (9.4%)	6,820 (9.6%)	1,701 (9.1%)	2,583 (9.4%)	813 (9.8%)	1,723 (10.3%)	27697 (9.5%)
Frailty and senility	8,802 (4.0%)	2,673 (3.8%)	751 (4.0%)	1,019 (3.7%)	236 (2.8%)	667 (4.0%)	11,475 (3.9%)
Cachexia	904 (0.4%)	441 (0.6%)	123 (0.7%)	231 (0.8%)	27 (0.3%)	60 (0.4%)	1,345 (0.5%)
Vitamin D deficiency	38,092 (17.2%)	14,575 (20.5%)	3,935 (21.1%)	5,233 (19.0%)	1,766 (21.2%)	3,641 (21.9%)	52,667 (18.0%)
Alcohol-abuse-related conditions	3,330 (1.5%)	1,094 (1.5%)	275 (1.5%)	491 (1.8%)	88 (1.1%)	240 (1.4%)	4,424 (1.5%)
Alcohol dependence, psychosis, etc.	2,859 (1.3%)	936 (1.3%)	233 (1.2%)	435 (1.6%)	64 (0.8%)	204 (1.2%)	3,795 (1.3%)
Polyneuropathy	181 (0.1%)	57 (0.1%)	14 (0.1%)	27 (0.1%)	5 (0.1%)	11 (0.1%)	238 (0.1%)

Features	Statin tolerant	Statin intolerant	Absolute statin intolerant		Partial statin intolerant		Total
			High confidence	Low confidence	High confidence	Low confidence	
	n = 221,442	n = 71,161	n = 18,652	n = 27,530	n = 8,318	n = 16,661	n = 292,603
Myopathy	0 (0%)	0 (0%)	0 (0%)	0 (0%)	0 (0%)	0 (0%)	0 (0%)
Pancreatitis	123 (0.1%)	45 (0.1%)	14 (0.1%)	21 (0.1%)	4 (0%)	6 (0%)	168 (0.1%)
Liver disease	484 (0.2%)	150 (0.2%)	43 (0.2%)	54 (0.2%)	18 (0.2%)	35 (0.2%)	634 (0.2%)
Hypothyroidism	19,512 (8.8%)	6,571 (9.2%)	1,697 (9.1%)	2,486 (9.0%)	792 (9.5%)	1,596 (9.6%)	26,083 (8.9%)
Liver disease	2,336 (1.1%)	871 (1.2%)	264 (1.4%)	326 (1.2%)	88 (1.1%)	193 (1.2%)	3,207 (1.1%)
CKD	10,102 (4.6%)	3,593 (5.0%)	993 (5.3%)	1,394 (5.1%)	376 (4.5%)	830 (5.0%)	13,695 (4.7%)
**Treatment usage**							
**Statins**							
Total	204,612 (92.4%)	63,328 (89.0%)	14,287 (76.6%)	26,401 (95.9%)	7,495 (90.1%)	15,145 (90.9%)	267,940 (91.6%)
Simvastatin	109,835 (49.6%)	28,390 (39.9%)	7,535 (40.4%)	12,719 (46.2%)	2,287 (27.5%)	5,848 (35.1%)	138,225 (47.2%)
Atorvastatin	81,712 (36.9%)	26,011 (36.6%)	5,521 (29.6%)	12,141 (44.1%)	4,068 (48.9%)	4,282 (25.7%)	107,723 (36.8%)
Rosuvastatin	5,757 (2.6%)	1,284 (1.8%)	37 (0.2%)	606 (2.2%)	208 (2.5%)	433 (2.6%)	7,041 (2.4%)
Others	7,308 (3.3%)	7,643 (10.7%)	1,194 (6.4%)	936 (3.4%)	932 (11.2%)	4,582 (27.5%)	14,951 (5.1%)
**Non-statins**							
Total	24,170 (10.9%)	16,924 (23.8%)	6,157 (33.0%)	1,540 (5.6%)	4,523 (54.4%)	4,820 (28.9%)	41,094 (14.0%)
Ezetimibe (Mono or in combination with statins)	21,291 (9.6%)	14,743 (20.7%)	4,031 (21.6%)	1,402 (5.1%)	4,490 (54.0%)	4820 (28.9%)	36,034 (12.3%)
Fenofibrate	1,329 (0.6%)	933 (1.4%)	933 (5.0%)	0 (0%)	0 (0%)	0 (0%)	2262 (0.8%)
Bezafibrate	1,107 (0.5%)	801 (1.2%)	746 (4.0%)	55 (0.2%)	0 (0%)	0 (0%)	1,908 (0.7%)
Others	443 (0.2%)	447 (0.7%)	448 (2.4%)	83 (0.3%)	33 (0.4%)	0 (0%)	890 (0.3%)
**SAMS**							
Total*	11,290 (5.1%)	4,902 (6.9%)	1,894 (10.2%)	1,067 (3.9%)	815 (9.8%)	1,126 (6.8%)	16,192 (5.5%)
Rhabdomyolysis	21 (0%)	13 (0%)	8 (0%)	4 (0%)	0 (0%)	1 (0%)	34 (0%)

Features	Statin tolerant	Statin intolerant	Absolute statin intolerant		Partial statin intolerant		Total
			High confidence	Low confidence	High confidence	Low confidence	
	n = 221,442	n = 71,161	n = 18,652	n = 27,530	n = 8,318	n = 16,661	n = 292,603
Other myositis	10 (0%)	9 (0%)	3 (0%)	4 (0%)	0 (0%)	2 (0%)	19 (0%)
Myalgia	4,240 (1.9%)	1,882 (2.6%)	800 (4.3%)	356 (1.3%)	330 (4.0%)	396 (2.4%)	6,122 (2.1%)
Myositis	76 (0%)	60 (0.1%)	31 (0.2%)	8 (0%)	9 (0.1%)	12 (0.1%)	136 (0%)
Drug-induced myopathy	16 (0%)	23 (0%)	15 (0.1%)	1 (0%)	3 (0%)	4 (0%)	39 (0%)
Other specified myopathies	50 (0%)	18 (0%)	8 (0%)	3 (0%)	2 (0%)	5 (0%)	68 (0%)
Myopathy due to other toxic agents	2 (0%)	2 (0%)	1 (0%)	0 (0%)	0 (0%)	1 (0%)	4 (0%)
Myopathy, unspecified	165 (0.1%)	130 (0.2%)	62 (0.3%)	18 (0.1%)	22 (0.3%)	28 (0.2%)	295 (0.1%)
Rheumatism, unspecified	2,543 (1.1%)	1,243 (1.7%)	336 (1.8%)	389 (1.4%)	193 (2.3%)	325 (2.0%)	3,786 (1.3%)
Cramps/spasms of the muscles	4,808 (2.2%)	1,864 (2.6%)	769 (4.1%)	330 (1.2%)	326 (3.9%)	439 (2.6%)	6,672 (2.3%)
**Other adverse events**							
Total*	64,606 (29.2%)	23,096 (32.5%)	6,207 (33.3%)	8,797 (32.0%)	2,731 (32.8%)	5,361 (32.2%)	87,702 (30.0%)
Arthralgia	811 (0.4%)	269 (0.4%)	81 (0.4%)	95 (0.3%)	34 (0.4%)	59 (0.4%)	1,080 (0.4%)
Constipation	12,121 (5.5%)	4,408 (6.2%)	1,224 (6.6%)	1,869 (6.8%)	427 (5.1%)	888 (5.3%)	16,529 (5.6%)
Diarrhea	293 (0.1%)	119 (0.2%)	41 (0.2%)	45 (0.2%)	7 (0.1%)	26 (0.2%)	412 (0.1%)
Abdominal pain	11,906 (5.4%)	4,750 (6.7%)	1,288 (6.9%)	1,773 (6.4%)	567 (6.8%)	1,122 (6.7%)	16,656 (5.7%)
Flatulence	4,181 (1.9%)	1,727 (2.4%)	511 (2.7%)	613 (2.2%)	197 (2.4%)	406 (2.4%)	5,908 (2.0%)
Nausea and vomiting	8,261 (3.7%)	3,450 (4.8%)	944 (5.1%)	1,422 (5.2%)	376 (4.5%)	708 (4.2%)	11,711 (4.0%)
Gastritis & duodenitis	30,214 (13.6%)	10,731 (15.1%)	2,863 (15.3%)	3,976 (14.4%)	1,328 (16.0%)	2,564 (15.4%)	40,945 (14.0%)
Anaphylaxis	5,840 (2.6%)	2,344 (3.3%)	608 (3.3%)	896 (3.3%)	299 (3.6%)	541 (3.2%)	8,184 (2.8%)
Rash and flushing	9,108 (4.1%)	3,397 (4.8%)	922 (4.9%)	1,293 (4.7%)	399 (4.8%)	783 (4.7%)	12,505 (4.3%)
Cognitive impairment	4,001 (1.8%)	1,323 (1.9%)	357 (1.9%)	509 (1.8%)	129 (1.6%)	328 (2%)	5,324 (1.8%)
**Drug–Drug**							
Itraconazole, posaconazole, miconazole	1,342 (0.6%)	522 (0.7%)	163 (0.9%)	177 (0.6%)	62 (0.7%)	120 (0.7%)	1,864 (0.6%)

Features	Statin tolerant	Statin intolerant	Absolute statin intolerant		Partial statin intolerant		Total
			High confidence	Low confidence	High confidence	Low confidence	
	n = 221,442	n = 71,161	n = 18,652	n = 27,530	n = 8,318	n = 16,661	n = 292,603
Erythromycin, telithromycin, clarithromycin	9,833 (4.4%)	3,615 (5.1%)	1,027 (5.5%)	1,425 (5.2%)	392 (4.7%)	771 (4.6%)	13,448 (4.6%)
Amprenavir, atazanavir, fosamprenavir, indinavir, lopinavir, nelfinavir, ritonavir, tipranavir	8 (0%)	10 (0%)	4 (0%)	2 (0%)	1 (0%)	3 (0%)	18 (0%)
Gemfibrozil	57 (0%)	67 (0.1%)	42 (0.2%)	10 (0%)	6 (0.1%)	9 (0.1%)	124 (0%)
Verapamil, diltiazem	3,649 (1.6%)	1,353 (1.9%)	381 (2.0%)	484 (1.8%)	156 (1.9%)	332 (2.0%)	5,002 (1.7%)
Warfarin	212 (0.1%)	66 (0.1%)	21 (0.1%)	27 (0.1%)	4 (0%)	14 (0.1%)	278 (0.1%)
Amiodarone	4,557 (2.1%)	1,596 (2.2%)	382 (2.0%)	550 (2.0%)	215 (2.6%)	449 (2.7%)	6,153 (2.1%)

ASCVD, atherosclerotic cardiovascular disease; CKD, chronic kidney disease; CV, cardiovascular; EMR, electronic medical record; ML, machine learning.

Table S8. Statin intolerance prevalence estimates based on EMR data (by risk factors).

**Prevalence estimates**	**Total universe**	**Statin tolerant**				**Statin intolerant by risk factor**			
		**Total**	**ASCVD**	**High CV risk**	**Hypercholesterolemia**	**Total**	**ASCVD**	**High CV risk**	**Hypercholesterolemia**
SI rules (EMR)<br>SI rules + supervised ML (EMR)	292,603 (100%)	224,112 (76.6%)	124,938 (55.7%)	43,918 (19.6%)	52,587 (23.5%)	71,161 (24.3%)	40,023 (56.2%)	17,224 (24.2%)	13,914 (19.6%)

ASCVD, atherosclerotic cardiovascular disease; CKD, chronic kidney disease; CV, cardiovascular; EMR, electronic medical record; ML, machine learning.
